# Whole-Genome Sequencing Analysis of Drug-Resistant *Salmonella Typhi* in Children

**DOI:** 10.3390/pathogens14100967

**Published:** 2025-09-24

**Authors:** Muhammad Riaz, Shabir Ahmad, Fazal Sattar, Ganwu Li, Zia Ud Din, Sajjad Ahmad, Azra, Aiman Waheed, Ihtisham Ul Haq, Jody E. Phelan, Gulab Fatima Rani, Otavio Cabral-Marques, Susana Campino, Taj Ali Khan, Taane G. Clark

**Affiliations:** 1 Department of Allied Health Sciences, Sarhad University of Science and Information Technology, Peshawar 25000, Pakistan; riazaurakzai@gmail.com (M.R.);; 2 National Institute for Biotechnology and Genetic Engineering College, Pakistan Institute of Engineering and Applied Sciences (NIBGE-C, PIEAS), Faisalabad 38000, Punjab, Pakistan; 3 Department of Veterinary Diagnostic and Production Animal Medicine, College of Veterinary Medicine, Iowa State University, Ames, IA 50011, USA; 4 Department of Pharmacy, Kohat University of Science and Technology, Kohat 26000, Pakistan; 5 Institute of Pathology and Diagnostic Medicines, Khyber Medical University, Peshawar 25100, Pakistanranigulabfatima@gmail.com (G.F.R.); 6 Centre of Biotechnology and Microbiology, University of Peshawar, Peshawar 25120, Pakistan; 7 Post-Graduate Programme in Innovation in Technology, Federal University of Minas Gerais, Belo Horizonte 30150-240, MG, Brazil; 8 Faculty of Infectious and Tropical Diseases, London School of Hygiene & Tropical Medicine, London WC1E 7HT, UK; 9 Department of Immunology, Institute of Biomedical Sciences, University of São Paulo, São Paulo 05508-220, SP, Brazil; 10 Laboratory of Medical Investigation 29, Division of Molecular Medicine, Department of Medicine, School of Medicine, University of São Paulo, São Paulo 05508-220, SP, Brazil; 11 Department of Clinical and Toxicological Analyses, School of Pharmaceutical Sciences, University of São Paulo, São Paulo 05508-220, SP, Brazil; 12 D’Or Institute for Research and Education (IDOR), São Paulo 01401-002, SP, Brazil; 13 Public Health Reference Laboratory, Khyber Medical University, Peshawar 25100, Pakistan; 14 Faculty of Epidemiology and Population Health, School of Hygiene and Tropical Medicine, London WC1E 7HT, UK

**Keywords:** *Salmonella Typhi*, antimicrobial resistance, whole-genome sequencing, beta-lactamase genes (*blaCTX-M-15*; *blaTEM-1B*), *gyrA-S83F* mutation, genetic resistome, plasmid-mediated resistance

## Abstract

Typhoid fever, caused by *Salmonella enterica* subsp. *enterica* serovar *Typhi* (*S. typhi*), remains a major public health concern, particularly in low-resource settings with poor sanitation. The emergence of multidrug-resistant (MDR) and extensively drug-resistant (XDR) strains have significantly complicated treatment, especially in vulnerable pediatric populations. This study aimed to characterize the genetic profiles of drug resistance in MDR and XDR *S. typhi* isolates from pediatric patients. Methods: A cross-sectional study was conducted on 800 blood samples from pediatric typhoid patients. *S. typhi* isolates were identified using the BacT/ALERT 3D system, followed by culture on MacConkey and blood agar. Antimicrobial susceptibility was assessed using the disk diffusion method according to CLSI 2022 guidelines. Whole-genome sequencing (WGS) was performed on 29 isolates using Illumina MiSeq technology, and resistance genes and mutations were analyzed. Results: Antimicrobial susceptibility testing revealed that 68 (48.57%) of *S. typhi* isolates were XDR and 61 (43.57%) were MDR, exhibiting widespread resistance to ciprofloxacin, ampicillin, chloramphenicol, ceftriaxone, and co-trimoxazole. WGS identified key resistance genes across all 29 isolates, including *bla*_CTX-M-15, *bla*_TEM-1B, *qnrS1*, *aac(6′)*-Iaa, *catA1*, *dfraA7*, *sul1*, *qacEΔ1*, and the *gyrA*-S83F mutation. Notably, *gyrA*-S83F and *qnrS1* were detected in all isolates and strongly correlated with ciprofloxacin resistance. Virulence genes were consistently present in all isolates, indicating a high pathogenic potential. The IncY plasmid, found in four (14%) isolates, was linked to resistance against third-generation cephalosporins, including ceftriaxone. Conclusion: This study underscores the alarming prevalence of MDR and XDR *S. typhi* isolates among pediatric patients, driven by resistance genes such as *bla*_CTX-M-15, *bla*_TEM-1B, and *gyrA*-S83F. These findings highlight the urgent need for targeted therapeutic strategies and robust surveillance systems to combat the growing threat of drug-resistant typhoid fever.

## 1. Introduction

Typhoid fever remains a significant public health challenge in developing regions, particularly in Asian countries [1]. This systemic illness, characterized by prolonged fever, abdominal pain, and gastrointestinal symptoms, is caused by *Salmonella Typhi* (*S. typhi*) [2]. *Salmonella* is a Gram-negative rod from the Enterobacteriaceae family, classified into serotypes based on the structures of its H and O surface antigens [3].

Globally, *S. typhi* causes an estimated 21.5 million infections and 216,510 deaths annually, with South and Southeast Asia experiencing the highest disease burden with over 10 million cases and 100,000 deaths reported in 2000 alone [4]. Pediatric populations bear a disproportionately high burden of *S. typhi* infections in these regions [5], exacerbated by poor hand hygiene, unsafe food practices, and limited access to clean drinking water [6,7]. The immature gut immune systems of young children further increase their susceptibility to *Salmonella* infections [8].

Antibiotic resistance in *S. typhi* has become a major clinical concern. Resistance to first-line antibiotics such as ampicillin, nalidixic acid, chloramphenicol, and trimethoprim/sulfamethoxazole significantly affect disease severity and treatment outcomes. Due to high toxicity and side effects, some antibiotics, including sulfamethoxazole and ciprofloxacin, are no longer prescribed for pediatric *S. typhi* infections [8].

The emergence of antibiotic-resistant *S. typhi* strains has made typhoid fever increasingly difficult to deal with, particularly in regions such as Peshawar [9]. Once effective first-line antibiotics ampicillin, chloramphenicol, and trimethoprim/sulfamethoxazole now face widespread resistance, leading to treatment failures [10,11] The rising morbidity and mortality associated with MDR and XDR *S. typhi* poses a serious global health challenge [12,13]. Addressing this crisis requires innovative strategies to control and mitigate its impact [14,15].

Antibiotic resistance in *S. typhi* is primarily driven by genetic mutations and adaptations affecting key physiological processes [16,17]. Several well characterized genes play crucial roles in resistance mechanisms. For beta-lactam resistance, the *bla*_TEM gene encodes beta-lactamase enzymes that hydrolyze beta-lactam antibiotics, such as ampicillin, rendering them ineffective [18,19]. For chloramphenicol resistance, the *catA1* gene produces chloramphenicol acetyltransferase, inactivating the antibiotic [20,21]. For trimethoprim resistance, mutations in the *dfrA* gene, which encodes dihydrofolate reductase, reduce trimethoprim’s binding affinity, lowering its efficacy [22]. Understanding these resistance mechanisms is essential for developing targeted treatment strategies and preventing the spread of drug-resistant *S. typhi* [23,24].

Despite extensive research on antibiotic-resistant *S. typhi* in Pakistan, limited studies have focused on pediatric populations particularly in high-burden cities like Peshawar, where children face an elevated risk of severe infection [25,26]. Given their immature immune systems and increased antibiotic exposure, children represent a critical group for understanding the evolution of resistance mechanisms [27]. This study investigates the genetic variations associated with antibiotic resistance in *S. typhi* isolates from pediatric patients in Peshawar. Using whole-genome sequencing (WGS) and advanced bioinformatics analyses, we aim to develop a comprehensive genetic profile of *S. typhi* in this high-risk population [28]. By identifying and characterizing resistance related mutations, this research will provide essential insights to inform public health initiatives and support the development of targeted therapeutic strategies to curb the spread of drug-resistant *S. typhi* [12,29].

This study aims to elucidate the genetic resistome of *S. typhi* in pediatric patients, characterize antimicrobial resistance profiles, and investigate plasmid-mediated resistance mechanisms.

## 2. Materials and Methods

### 2.1. Study Design and Sampling

A cross-sectional study was conducted at Rehman Medical Institute, Peshawar, Khyber Pakhtunkhwa, Pakistan, to obtain *Salmonella* isolates. A total of 800 blood samples were collected from outpatient and inpatient departments following standard protocols. The BacT/ALERT 3D microorganism detection system (BioMérieux, Marcy l’Étoile, France) was used for initial screening. Positive samples were subcultured on MacConkey (Oxoid, Basingstoke, UK) and blood agar (Oxoid, UK) plates, incubated aerobically at 37 °C for 18–24 h. Bacterial identification was performed using standard laboratory procedures, including colony morphology, Gram staining, and the API 20E identification system (BioMérieux, France). The disc diffusion method was employed to assess *S. typhi* for Extended Spectrum Beta-Lactamase (ESBL) production. Mueller–Hinton agar [MHA (Oxoid, UK)] plates were inoculated with a 0.5 McFarland standard bacterial suspension, followed by the placement of cefotaxime (30 µg), ceftazidime (30 µg), and their combinations with clavulanic acid (30 µg/10 µg). After incubation at 37 °C for 18–24 h, inhibition zones were measured. An increase of ≥5 mm in zone diameter with the antibiotic clavulanic acid combination was considered indicative of ESBL production.

### 2.2. Phenotypic Antimicrobial Susceptibility Analysis

Antibiotic susceptibility testing (AST) was performed using the Kirby Bauer disc diffusion method. Bacterial colonies were subcultured on Mueller–Hinton agar and incubated at 37 °C for 24 h. The following antibiotic discs were used: imipenem (10 μg), azithromycin, ampicillin (10 μg), trimethoprim (5 μg), ciprofloxacin (5 μg), cefotaxime (5 μg), and chloramphenicol (30 μg). Results were interpreted according to the 2022 Clinical and Laboratory Standards Institute (CLSI) guidelines [30].

### 2.3. Whole Genomic Sequencing

Of the 140 culture-positive isolates, 31 were selected for WGS based on antibiotic resistance profiles and gender distribution. Genomic DNA was extracted from 31 *S. typhi* isolates using High Pure Viral Nucleic Acid Kit (catalog number 11858874001; Roche, Basel, Switzerland). DNA quality and quantity were assessed using a NanoDrop (Berthold Detection System GmbH, Pforzheim, Germany) and only 2 samples had poor quality and so were not further proceeded for WGS. A subset of 29 isolates was selected for WGS based on AST results and gender distribution. The sequencing of DNA samples on an Illumina MiSeq platform (2 × 150 bp chemistry) was facilitated by The Applied Genomics Centre, London School of Hygiene and Tropical Medicine (LSHTM), London, UK.

### 2.4. Data Analysis

The raw sequencing reads were processed following the previously published protocol [31]. Briefly, quality assessment was performed using FastQC (v0.12.1) and MultiQC (v1.19), and reads were trimmed with Trimmomatic (v0.36) to remove adapters and low-quality sequences. The cleaned reads were re-evaluated to ensure quality improvement. High-quality reads were assembled using SPAdes (v3.15.5), with small and low coverage contigs removed [32,33]. Contamination was assessed with CheckM2 [34], and any contaminated samples were excluded. Assembly quality was evaluated using QUAST (v3.9), and genome annotation was performed with Prokka (v1.14.6) [35,36] and BVBRC. Serotyping was conducted using SeqSero2 (v1.2.1) [37], while genotyping was determined with Pathogen Watch and the Genotyphi tool [38]. Geographic variation among *S. typhi* isolates was examined using the Bacterial Codon Tree tool from BVBRC. Two genome groups were analyzed: one consisting of 29 in house genomes and the other including 19 publicly available genomes collected between 2017 and 2021 from India, China, and Bangladesh.

Antibiotic resistance and virulence associated genes were identified using ABRicate (v1.0.1) (https://github.com/tseemann/abricate) with ResFinder and the Virulence Factor Database (VFDB) as references (accessed on 16 February 2024), applying a 90% threshold for coverage and identity [39]. Biocide resistance genes and quinolone resistance mutations were detected using AMRFinderPlus, targeting *Salmonella* spp. with the same parameters [40]. Sequence types (STs) were assigned using the PubMLST database and analyzed with MLST (v2.23.0) [41]. Plasmid replicons were identified using PlasmidFinder within ABRicate. To explore the genetic relationships between the sequenced *S. typhi isolates*, a core genome SNP phylogenetic tree was constructed using Parsnp (v2.0.3) [42] and metadata visualization was performed using iTOL [43]. The relationship between antibiotic resistance and factors such as age, gender, hospitalization status, and microbiological pathogen was examined using multivariate linear regression.

### 2.5. Patient Demographic and Clinical Data

Demographic data (age, gender, hospitalization status) and clinical parameters (Hb, TLC, ALT, CRP) were collected and analyzed.

### 2.6. Statistical Analysis

Data were analyzed using Stata (Version 16). Mann–Whitney U test and Kruskal–Wallis H test was applied to assess variability in antibiotic resistance. Multivariate linear regression was used to explore relationships between resistance and patient variables. A *p*-value < 0.05 was considered significant.

## 3. Results

### 3.1. Demographic Data

The study population consisted primarily of children aged 1 to 20 years, with a mean age of 8.93 years (±5.61 years). Among the 800 samples collected, 140 (17.5%) tested positive for *S. typhi* infection. Of these cases, 96 (68.57%) were male, highlighting a higher prevalence of infection among males in the study cohort (Table 1).

### 3.2. Hematological and Biochemical Parameters of the Infected Patients

Clinical evaluations of *S. typhi* patients revealed notable hematological and biochemical findings (Table 1). The mean hemoglobin (Hb) level was 11.09 g/dL (±1.49 g/dL), ranging from 7.2 to 16.4 g/dL. The mean total leukocyte count (TLC) was 6.56 × 10^3^/µL (±2.18 × 10^3^/µL), with values spanning 2.57 to 15.65 × 10^3^/µL, remaining within the normal reference range of 4.50 to 13.50 × 10^3^/µL. Alanine transaminase (ALT) levels exhibited substantial variability, with a mean of 76.83 U/L (±109.12 U/L), ranging from 13 to 855 U/L—well above the reference range of 5 to 50 U/L. Similarly, C-reactive protein (CRP) levels showed significant variation, with a mean of 10.80 mg/dL (±10.78 mg/dL), ranging from 0.59 to 57.2 mg/dL, considerably exceeding the normal reference range of 0.0 to 0.5 mg/dL.

### 3.3. Clinical Biomarkers and Age

The variation in clinical parameters with respect to age was analyzed among infected patients. Hb levels differed significantly across age groups. Children aged 1–7 years had significantly lower Hb levels than those aged 8–14 years (*p* < 0.0001) and 15–20 years (*p* < 0.0001). However, there was no significant difference in Hb levels between the 8–14 and 15–20-year-old groups (Supplementary Figure S1). A positive correlation was observed between age and Hb levels (R^2^ = 0.1907), suggesting that Hb levels tend to rise with age, though the association is relatively weak (Supplementary Figure S2). In contrast, TLC, Alanine ALT, and C-CRP showed no significant differences between age groups, despite overall *p*-values of 0.0046, 0.328, and 0.774, respectively (Supplementary Figure S1). Similarly, their correlations with age were weak, with R^2^ values of 0.078 (TLC), 0.0073 (ALT), and 0.009 (CRP), as shown in the scatter plots (Supplementary Figure S2).

### 3.4. Phenotypic Antibiotic Susceptibility Profiles of S. typhi Isolates

The results from phenotypic antibiotic susceptibility testing are summarized (Supplementary Figure S3). We found that most isolates exhibited complete 140 (100%) resistance to ciprofloxacin, rendering it ineffective against the tested strains. Ampicillin resistance was also high at 136 (97.14%), significantly limiting its clinical utility. Similarly, 125 (89.29%) of isolates were resistant to chloramphenicol, leaving only a small proportion susceptible. Ceftriaxone resistance was observed in 121 (86.43%) of cases, further reducing treatment options. Co-trimoxazole had a lower resistance rate of 68 (48.57%), indicating that just over half of the isolates remained susceptible. In contrast, azithromycin and meropenem showed no resistance, making them the most effective antibiotics against the tested strains. XDR patterns were prevalent, with 68 (48.57%) of isolates classified as XDR the largest proportion observed. MDR isolates accounted for 61 (43.57%) of cases, while only 11 (7.86%) of isolates were fully susceptible to the tested antibiotics.

### 3.5. Analysis of Sequenced S. typhi Isolates

**Figure 1:**
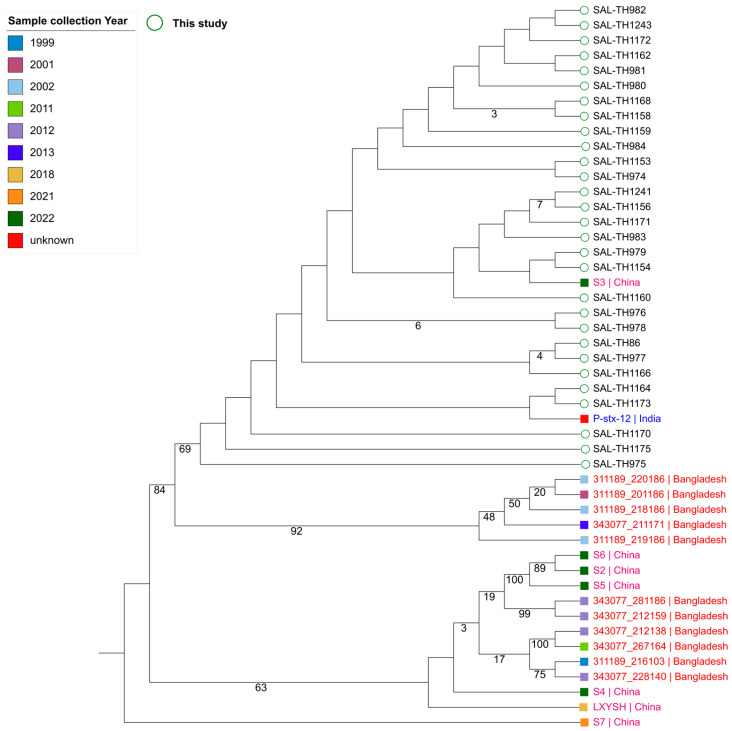
The phylogenetic analysis of *S. typhi* isolates from this study with those available in BVBRC from 2001 to 2022 from adjacent neighboring countries.

This study selected 31 *Salmonella Typhi* isolates for WGS. Biochemical testing confirmed their identity, but two isolates were identified as non-*S. typhi* and excluded from downstream analysis. Among the remaining 29 confirmed *S. typhi* isolates, 19 were from male patients and 10 from female patients. In silico serotyping it revealed that the isolates exhibited O antigen type 9, H1 antigen type “d,” and lacked the H2 antigen confirming their classification as *S. typhi*, a characteristic profile of this serotype. Multi-locus sequence typing (MLST) identified all isolates as sequence type 1 (ST1), belonging to genotype 4.3.1.1.P1, consistent with global patterns. Phylogenetic analysis supported the MLST clustering and revealed that two isolates (SAL-TH1164, SAL-TH1173) shared strong genetic similarities with strains from India, suggesting a common ancestor or similar evolutionary pressures (Figure 1). Additionally, genetic similarities between isolates SAL-TH1154 and SAL-TH979 and strains from China indicate potential past or ongoing transmission between China and Pakistan.

### 3.6. Antimicrobial Resistance Genes in the Sequenced S. typhi Isolates

**Figure 2:**
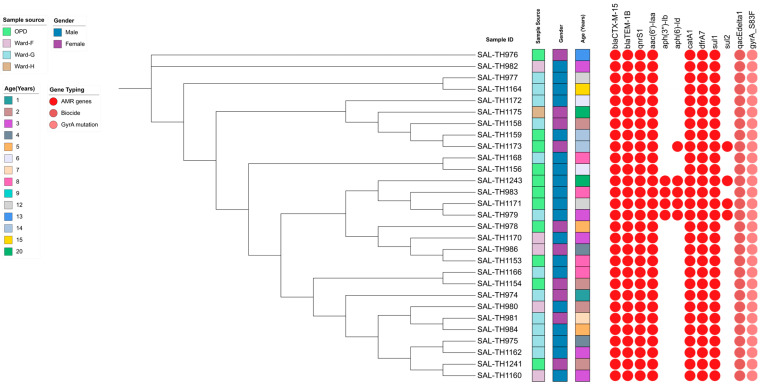
Distribution of antimicrobial and biocide resistance genes in the sequenced *S. typhi* isolates from this study.

Antimicrobial susceptibility testing classified 21 isolates as XDR and 8 as MDR. All XDR *S. typhi* strains were resistant to cell wall synthesis inhibitors (ampicillin, ceftriaxone), protein synthesis inhibitors (chloramphenicol), folate inhibitors (co-trimoxazole), and DNA replication inhibitors (ciprofloxacin). However, all clinical isolates remained susceptible to meropenem and azithromycin. The distribution of AMR genes across the 29 sequenced isolates revealed that *bla*_CTX-M-15, bla_TEM-1B, *qnrS1*, *aac(6′)*, *catA1*, *dfrA7*, *sul1*, *qacEΔ1*, and *gyrA*-S83F were present in 100% of the samples, indicating their widespread and consistent presence (Figure 2). In contrast, the aminoglycoside resistance genes *aph(3′)*-Ib and *aph(6)*-Id were detected in 13.7% and 17.2% of isolates, respectively, suggesting a lower but notable level of dissemination. Additionally, alongside *sul1*, the *sul2* gene also associated with aminoglycoside resistance was found in 14% of the samples.

A key point mutation associated with antibiotic resistance was detected in the *gyrA* gene of fluoroquinolone-resistant isolates (Supplementary Figure S4). The S83F mutation was present in all sequenced *S. typhi* isolates in this study.

**Figure 3:**
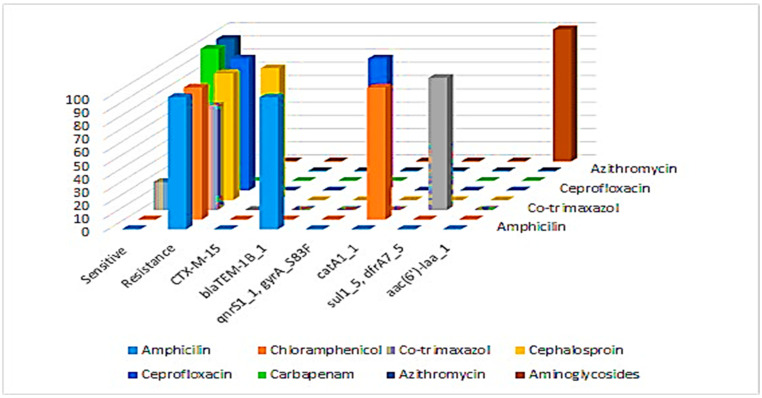
Correlation between genetic markers and antibiotic resistance in *S. typhi*.

Our study found a strong correlation between specific resistance genes and phenotypic antibiotic resistance patterns. The *bla*_TEM-1B gene was linked to 100% resistance to ampicillin, while the *bla*_CTX-M-15 gene was associated with 96% resistance to cephalosporins. The *gyrA*_S83F mutation and *qnrS1* gene correlated with 100% ciprofloxacin resistance, and the *catA1* gene was linked to complete (100%) resistance to chloramphenicol. Resistance to co-trimoxazole was associated with the *sul1*, *sul2*, and *dfrA7* genes and was observed in 79% isolates. The *aac(6′)*-Iaa gene was identified as genotypically linked to aminoglycoside resistance, though phenotypic testing was not performed. All isolates remained 100% susceptible to carbapenems and azithromycin, consistent with the absence of key resistance genes (Figure 3).

To investigate the genetic environment of β-lactamases, particularly the *bla*_CTX-M-15 ESBL gene responsible for ceftriaxone resistance, we analyzed the flanking regions. All isolates exhibited the same genetic architecture, except for isolate SAL-TH1243, which had a distinct genetic backbone.

In both groups, the flanking composite transposon contained the *bla*_CTX-M-15 gene at its center. In all isolates except SAL-TH1243, *bla*_CTX-M-15 was located on the same contig as the *bla*_TEM-1B gene, which confers ampicillin resistance. BLASTn analysis confirmed that this structure was identical across all isolates, except for the variation observed in SAL-TH1243 (Supplementary Figure S5).

### 3.7. Virulence Gene Profile of the Sequenced S. typhi Isolates

All analyzed bacterial samples exhibited a consistent and comprehensive virulence profile, with key virulence genes present in 100% cases. Genes involved in curli fiber formation (*csgA*, *csgB*, *csgC*, and *csgD*), essential for biofilm formation and surface adherence, were universally present. Similarly, type 1 fimbriae components (*fimA*, *fimC*, *fimD*, *fimF*, *fimH*, and *fimI*) were detected in all isolates, highlighting a strong capacity for host tissue adhesion. Hemolysin production genes (*hlyA*, *hlyB*, *hlyC*, and *hlyD*), which contribute to tissue damage and infection spread, were present in every sample. Toxin secretion genes (*vexA*, *vexB*, *vexC*, and *vexD*) were also consistently found, indicating a common mechanism for toxin delivery into host cells. P fimbriae genes (*papA*, *papC*, *papE*, *papF*, and *papG*), associated with urinary tract infections, and type 3 fimbriae genes (*mrkA*, *mrkB*, *mrkC*, and *mrkD*), linked to biofilm formation, were present in all isolates, reinforcing their role in bacterial colonization and environmental persistence. Additionally, iron acquisition genes (*fyuA*, *irp2*, and *ybtS*), crucial for bacterial survival in host environments, were universally detected. Notably, the *rpoS* gene, which regulates stress response, was absent in one isolate, suggesting minor variability in stress adaptation mechanisms. Overall, these findings indicate a highly virulent bacterial population well equipped for adhesion, invasion, and survival in host environments (Supplementary Figure S6).

### 3.8. Detection of Heavy Metal Resistance Genes in the Sequenced Isolates

The analysis of heavy metal resistance genes revealed a high degree of uniformity across the samples, indicating widespread resistance potential among the studied organisms. Several genes associated with zinc resistance were present in all samples, underscoring their importance in metal ion management. These include *mdtB* (encoding a transporter for metals and antimicrobial compounds), *pitA* (a phosphate transporter that reduces intracellular metal accumulation), *zupT/ygiE* (essential for zinc transport and homeostasis), *modC* (a molybdate transporter that regulates toxic concentrations), *acrD* (an efflux pump enhancing overall metal resistance), *pmrG* (which confers resistance to polymyxins and metals), *cpxA* (a sensor kinase responding to environmental stress), *fabI* (vital for bacterial survival), *pstS* (a phosphate-binding protein), *rcnR/yohL* (a regulator for nickel and cobalt), mntH/yfeP (involved in manganese transport), and the magnesium transporters *corA*, *corB*, and *mgtA* (critical for cellular stability). Additionally, *zraP* and *zntR/yhdM* were linked to zinc and lead resistance. The *corC* gene, associated with magnesium and cobalt resistance, was detected in 58.6% of the samples. The *robA* gene, which enhances stress resistance, was present in 44.8% of the samples, suggesting selective advantages in specific conditions. Conversely, *merA*, responsible for mercury resistance, was found in only 13.8% of the samples, indicating that mercury resistance is less critical in these environments, likely due to limited exposure (Supplementary Figure S7).

### 3.9. Plasmid Distribution and Correlation of IncY Plasmid on Cephalosporin Resistance

WGS of *S. typhi* isolates revealed that 14% of the samples contained the IncY plasmid, while 86% lacked it. Both phenotypic and genotypic evidence support the role of the IncY plasmid in promoting resistance, contributing to the emergence of XDR strains, particularly in Pakistan.

## 4. Discussion

Typhoid fever remains a significant public health issue, especially in developing regions such as Pakistan, where access to sanitation and clean water is limited. This study aimed to analyze 800 blood samples collected from patients at Rehman Medical Institute, Peshawar, Pakistan. Clinical parameters, including Hb levels, TLC, ALT, and CRP levels, were measured alongside phenotypic antibiotic susceptibility profiles. Based on the phenotypic antibiotic susceptibility results and gender, a subset of 29 samples was selected for WGS to identify antibiotic resistance genes, point mutations associated with antimicrobial resistance, virulence factors, and plasmid replicons.

Our study found that children, with an average age of 8.9 years, had a higher frequency of *S. typhi* infections, which is consistent with other studies [44,45,46]. A study in South India also reported a higher frequency of *S. typhi* infection in this age group [47]. Hematological and biochemical analyses showed that the average hemoglobin level was slightly below the normal reference range (11.5–15.5 g/dL), indicating mild anemia among the population. While TLC levels were within the normal range, elevated ALT and low CRP values suggested potential liver dysfunction. The combined decrease in hemoglobin levels and elevated ALT and CRP indicate mild anemia with hepatic involvement. Age correlated with increased Hb levels, while no meaningful correlation was observed between other clinical parameters (TLC, ALT, and CRP) and age. These findings align with previous research on clinical manifestations in typhoid patients [48].

Phenotypic antibiotic susceptibility testing revealed significant resistance in our isolates to ampicillin, chloramphenicol, ceftriaxone, and ciprofloxacin. These results are consistent with prior studies documenting resistance to these antibiotics [49,50]. Interestingly, the isolates remained susceptible to azithromycin and meropenem, indicating their potential role in treating resistant strains. This aligns with updated treatment guidelines that recommend the use of these antibiotics for resistant *S. typhi* strains [51].

WGS of the 29 isolates identified a broad array of antibiotic resistance genes against first- and second-line antibiotics. All isolates carried resistance genes such as *bla*_CTX-M-15, *bla*_TEM-1B, *qnrS1*, *aac*(*6′*)-Ib, *catA1*, *dfrA7*, and *sul1*. Additionally, 14% of the isolates also contained the *sul2* gene, conferring resistance to sulfonamides. The widespread presence of these resistance genes indicates a significant spread of resistance traits among *S. typhi* isolates in the region, consistent with findings from other local studies [52]. Mutations in *gyrA*, *gyrB*, *parC*, and *parE* also contributed to fluoroquinolone resistance. Previous research has shown that resistance to nalidixic acid and ciprofloxacin is often linked to multiple mutations in *gyrA*, *gyrB*, *parC*, and *parE* genes, but no additional mutations were observed in our samples. The S83F mutation involves the substitution of serine (Ser) with phenylalanine (Phe) at position 83 (Ser83Phe), replacing a small polar amino acid with a bulkier, nonpolar residue. Serine’s hydroxyl group facilitates protein flexibility and polar interactions, whereas phenylalanine increases hydrophobicity and may introduce steric hindrance. These structural changes likely disrupt normal protein interactions, contributing to fluoroquinolone resistance in *S. typhi*. Notably, all isolates carried the S83F mutation in *gyrA*, which has been previously reported as a key mutation for quinolone resistance in *S. typhi* [53,54].

The presence of the IncY plasmid in 14% of the isolates is concerning, particularly due to its association with resistance to third-generation cephalosporins like ceftriaxone. Reduced susceptibility to fluoroquinolones and resistance to third generation cephalosporins and azithromycin have also been reported [55]. This study underscores the critical role of plasmid-mediated resistance in the spread of resistance traits. The IncY plasmid carries resistance genes that can be transferred between bacterial strains via horizontal gene transfer, promoting the rapid spread of resistance within bacterial populations. Plasmids, which often carry genes associated with antibiotic resistance and virulence, play a crucial role in bacterial adaptability. The presence of the IncY plasmid in a minority of isolates suggests a potential for increased resistance or pathogenicity. The IncY plasmid is particularly significant due to its association with antibiotic resistance, especially to third-generation cephalosporins like ceftriaxone. This plasmid commonly harbors ESBL genes, such as *bla*_CTX-M, which degrade beta-lactam antibiotics and complicate treatment strategies. Phenotypic confirmation of ESBL production was observed in strains carrying the IncY plasmid, which aligns with the genotypic findings that indicate the plasmid harbors genes responsible for this resistance. Other studies have highlighted the emergence of globally dominant plasmids, such as IncHI1, associated with multiple drug-resistant typhoid [56]. The ability of the IncY plasmid to disseminate resistance genes across strains and species positions it as a major factor in the development and persistence of multidrug-resistant pathogens [57]. Given its role in reducing the efficacy of critical antibiotics such as ceftriaxone, monitoring the prevalence of plasmids like IncY is essential for informing antibiotic stewardship efforts and guiding strategies to control plasmid-mediated resistance. These findings align with other research emphasizing the need for targeted public health interventions to mitigate the spread of antibiotic-resistant pathogens [58].

## 5. Conclusions

The findings of this study highlight the urgent need for enhanced treatment protocols and antibiotic stewardship initiatives to combat the rising threat of MDR and XDR *S. typhi* in Pakistan. By addressing these critical gaps, our findings will contribute to a deeper understanding of drug-resistant *S. typhi* and support more effective strategies for its management and control in Peshawar and beyond. Given the widespread presence of resistance genes and the potential for plasmid-mediated gene transfer, it is critical to implement targeted immunization campaigns, explore alternative treatment options, and establish continuous surveillance to prevent the further spread of resistant strains. This distribution highlights a significant challenge in managing typhoid infections, with most cases exhibiting resistance to multiple or extensive drugs. Future research should prioritize understanding the mechanisms underlying the acquisition of resistance genes and developing novel therapeutic strategies. These findings are pivotal for improving treatment strategies, particularly for pediatric typhoid fever patients, by identifying key resistance-conferring mutations. These findings highlight the cross-border movement of *Salmonella* strains between Pakistan, India, and China, emphasizing the need for regional collaboration in controlling typhoid and other infectious diseases. Additionally, the genetic profiles generated in this study will support the development of tailored vaccination campaigns and antimicrobial stewardship programs specific to the epidemiological context of Peshawar. In turn, this will contribute to a more effective public health response to the challenges posed by drug-resistant *S. typhi*, enabling better prevention and control efforts.

## Figures and Tables

**Table 1:** Important clinical parameters of 800 infected individuals.

Variables	Mean	SD	Min–Max	Ref. Value
Age (year)	8.93	5.61	1–20	-
Hb (g/dL)	11.09	1.49	7.2–16.4	11.5–15.5
TLC (10^3^/µL)	6.56	2.18	2.57–15.65	4.50–13.50
ALT (U/L)	76.83	109.12	13–855	5–50
CRP (mg/dL)	10.80	10.78	0.59–57.2	0.0–0.5

SD standard deviation; Min minimum; Max maximum; hemoglobin Hb; total leukocyte counts TLC; alanine transaminase ALT; C-reactive protein CRP.

## Data Availability

The original contributions presented in the study are included in the article/Supplementary Material, further inquiries can be directed to the corresponding authors.

## Supplementary Materials

The following supporting information can be downloaded at: https://www.mdpi.com/article/10.3390/pathogens14100967/s1, Figure S1: Age-related differences in Hemoglobin (Hb), total leukocyte count (TLC), Alanine transaminase (ALT), and C-reactive protein (CRP) Levels were observed in the studied samples; Figure S2: Correlation between Age and the Hemoglobin (Hb), total leukocyte count (TLC), Alanine transaminase (ALT), and C-reactive protein (CRP) Levels; Figure S3: Phenotypic Antibiotic Susceptibility pattern of the isolates from this study; Figure S4: The 3D protein structure of wild and mutated protein depicting the effect of the mutation on the function; Figure S5: Two different types of genetic backbone structure found around the ceftriaxone resistance blaCTX-M-15 gene in the sequenced isolates; Figure S6: Virulence gene profiles of the sequenced *S. Typhi* isolates from this study; Figure S7: Phylogenetic Distribution and Heavy Metal Resistance Gene Profiles of *S. Typhi* Isolates.
