# Supplementary material for: Whole-Genome Sequencing Analysis of Drug-Resistant Salmonella Typhi in Children
Source: Pathogens. 2025 Sep 24;14(10):967. doi: 10.3390/pathogens14100967 (PMC12566932; doi:10.3390/pathogens14100967)
Supplement: Supplementary file 1 [file pathogens-14-00967-s001.zip › pathogens-3872985-supplementary.pdf]

SUPPLEMENTARY INFORMATION

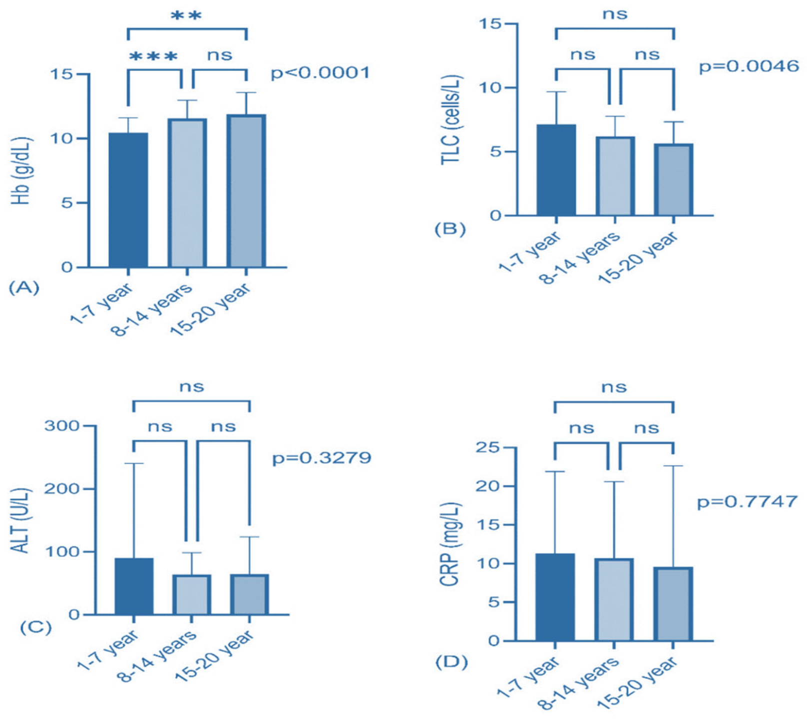

Figure S1. Age-related differences in Hemoglobin (Hb), total leukocyte count (TLC), Alanine transaminase (ALT), and C-reactive protein (CRP) Levels were observed in the studied samples.

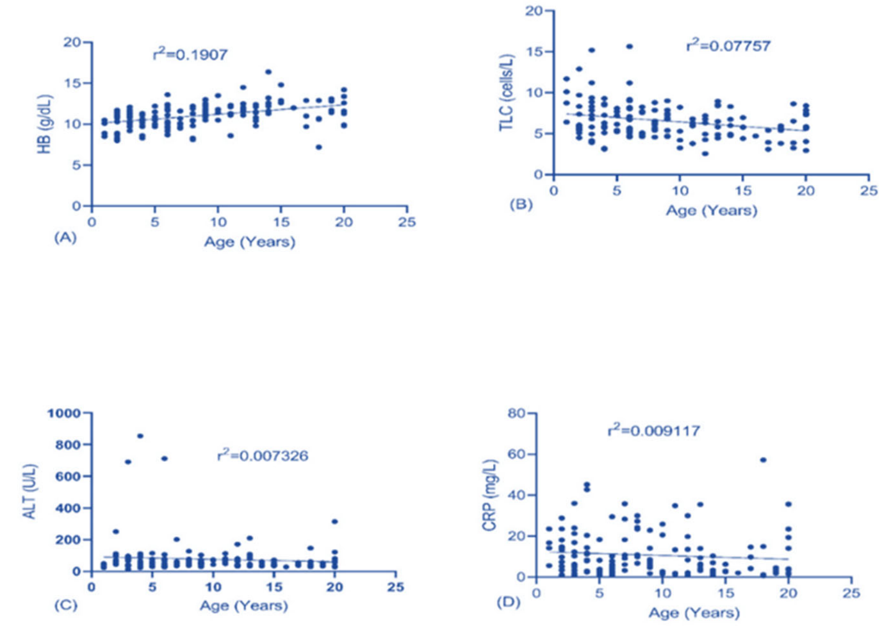

Figure S2. Correlation between Age and the Hemoglobin (Hb), total leukocyte count (TLC), Alanine transaminase (ALT), and C-reactive protein (CRP) Levels.

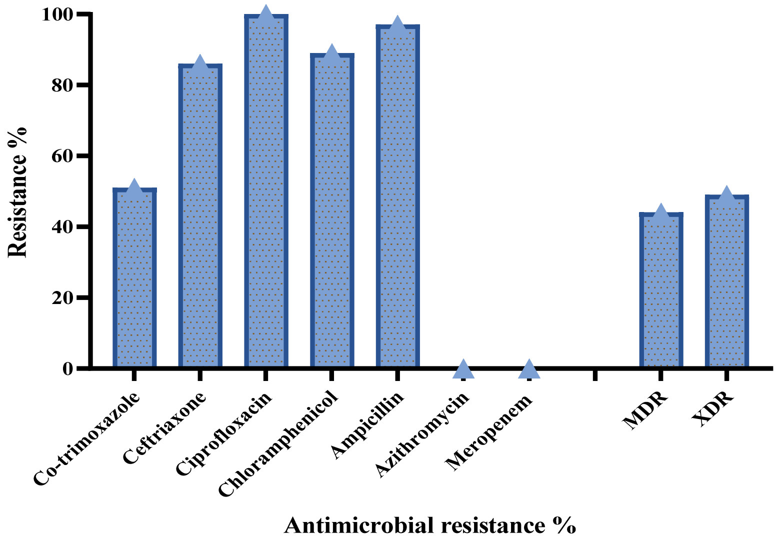

Figure S3. Phenotypic Antibiotic Susceptibility pattern of the isolates from this study

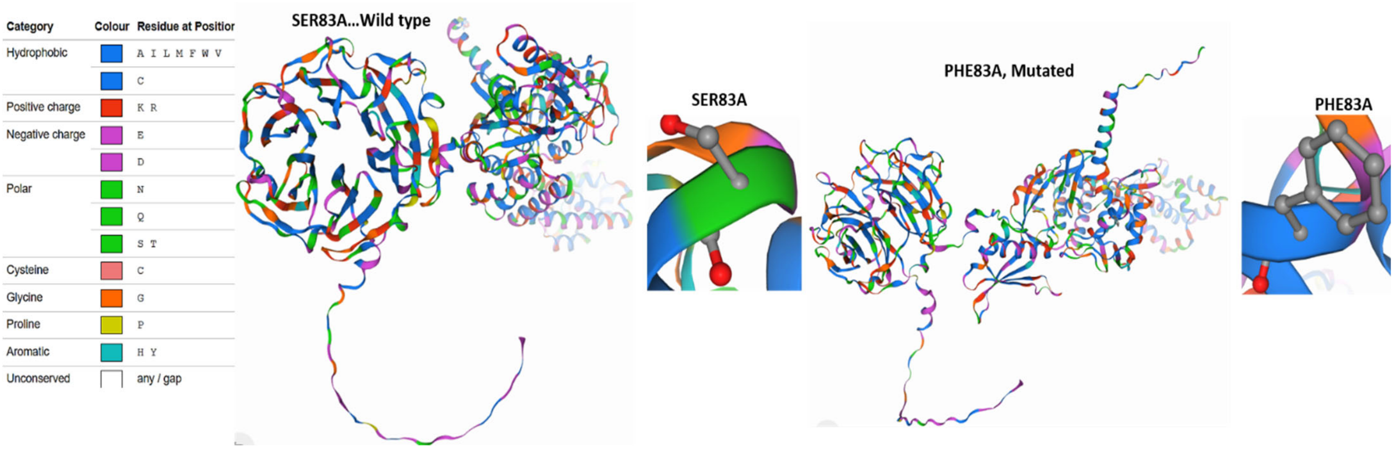

Figure S4. The 3D protein structure of wild and mutated protein depicting the effect of the mutation on the function.

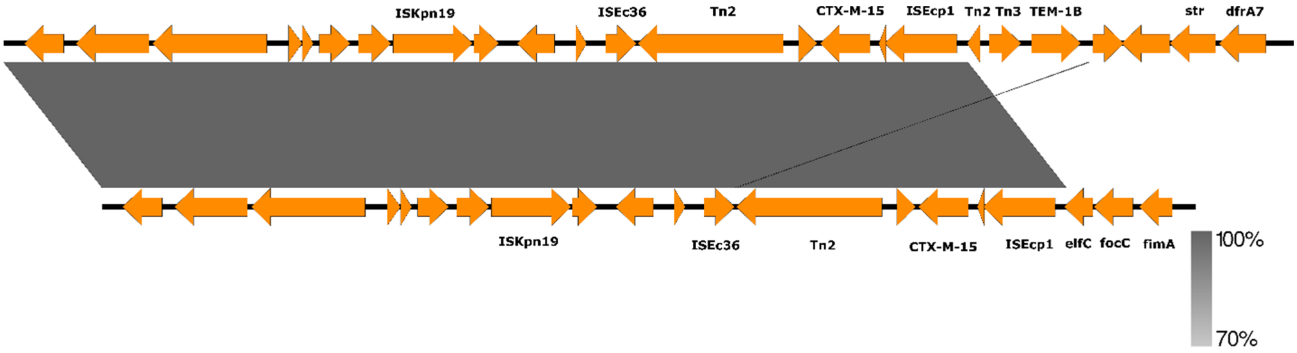

Figure S5. Two different types of genetic backbone structure found around the ceftriaxone resistance *bla*$_{CTX\text{-}M\text{-}15}$ gene in the sequenced isolates.

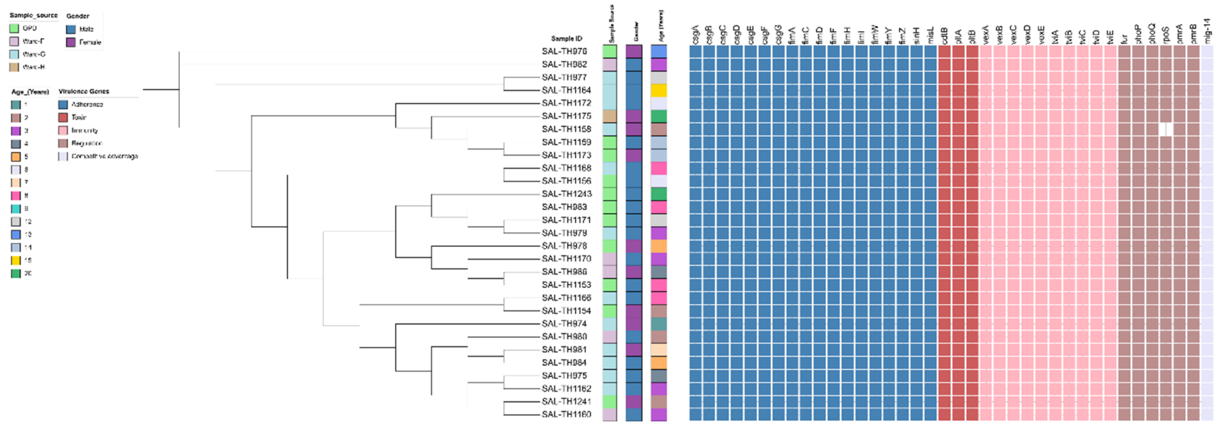

Figure S6. Virulence gene profiles of the sequenced *S. Typhi* isolates from this study.

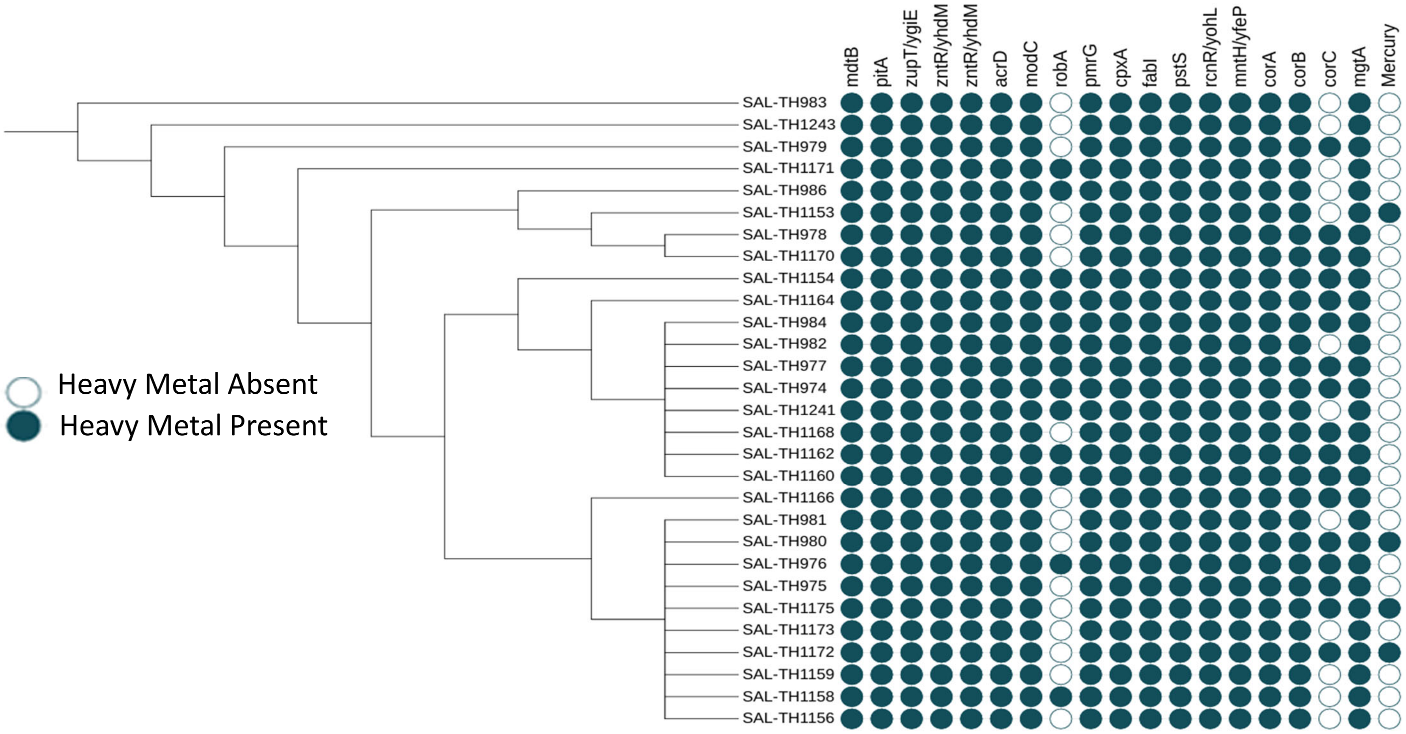

Figure S7. Phylogenetic Distribution and Heavy Metal Resistance Gene Profiles of *S. Typhi* Isolates
